# The Liability of Hospital Committees under the Workmen's Compensation Act, 1906

**Published:** 1907-03-02

**Authors:** Chas. W. Dickinson

**Affiliations:** Secretary. Royal Albert Hospital, Devonport


### THE LIABILITY OF HOSPITAL COMMITTEES UNDER THE WORKMEN'S COMPENSATION ACT, 1906.

*To the Editor of* The Hospital.

Sir,—As, doubtless, has been the case with the governing bodies of all hospitals throughout the Kingdom, the Managing Committee of this Institution has had under consideration the question above referred to, and they would be glad to know whether the responsible authorities of the large Metropolitan or Provincial hospitals intend taking concerted action in this connection.

Apart from the question of liability to domestic servants under the new Act, the existence of which there would seem to be but little doubt, the far weightier question arises as to a hospital committee's responsibility, under that Act, in regard to salaried servants, such as the resident medical officers, the nursing staff, whether employed in the hospital or in nursing cases outside, the dispensers and their assistants, and the office staff.

May I ask you, Sir, kindly to find space in your valued columns for this letter, and to invite the opinion of experts in such matters on the important subject to which it relates.

I am, dear Sir, yours faithfully,

Chas. W. Dickinson, Secretary.

Royal Albert Hospital, Devonport, February 25, 1907.

